# Directional picoantenna behavior of tunnel junctions formed by an atomic-scale surface defect

**DOI:** 10.1126/sciadv.adn2295

**Published:** 2024-09-25

**Authors:** David Mateos, Oscar Jover, Miguel Varea, Koen Lauwaet, Daniel Granados, Rodolfo Miranda, Antonio I. Fernandez-Dominguez, Alberto Martin-Jimenez, Roberto Otero

**Affiliations:** ^1^ IMDEA Nanociencia, Madrid, Spain.; ^2^ Departamento de Física de la Materia Condensada and Condensed Matter Physics Center (IFIMAC), Universidad Autónoma de Madrid, Madrid, Spain.; ^3^ Departamento de Física Teórica de la Materia Condensada and Condensed Matter Physics Center (IFIMAC), Universidad Autónoma de Madrid, Madrid, Spain.

## Abstract

Plasmonic nanoantennas have attracted much attention lately, among other reasons because of the directionality of light emitted by fluorophores coupled to their localized surface plasmon resonances. Plasmonic picocavities, i.e., cavities with mode volumes below 1 nm^3^, could act as enhanced antennas due to their extreme field confinement, but the directionality on their emission is difficult to control. In this work, we show that the plasmonic picocavity formed between the tip of a scanning tunneling microscope and a metal surface with a monoatomic step shows directional emission profiles and, thus, can be considered as a realization of a picoantenna. Electromagnetic calculations demonstrate that the observed directionality arises from the reshaping and tilting of the surface charges induced at the scanning tip due to the atomic step. Our results pave the way to exploiting picoantennas as an efficient way for the far-field probing and control of light-matter interactions below the nanoscale.

## Teaser

Atomic defects at STM junctions create picoantennas with directional emission.

## INTRODUCTION

Manipulation of light at subwavelength scales can be accomplished by leveraging the extreme confinement of electromagnetic (EM) fields by metallic nanostructures, supporting localized surface plasmon (LSP) resonances. The size and shape of the nanostructure ultimately determine the near-field interaction between LSPs and nanoscale light sources, such as atoms, molecules, or quantum dots, which in turn dictates the radiated power and spectral and angular distributions of the emitted light in the far field (*1*–*4*). In particular, dielectric gaps are a fundamental element in engineered metallic nanostructures, which can be designed to shape the emission of fluorophores they host, and, in this context, they are usually termed nanoantennas. So far, control over the directionality of light has been achieved at the scale of several nanometers. Different strategies involving single nanoparticles (*5*–*7*) and nanoparticle arrangements (*8*–*14*) have been proposed to manipulate the directionality of light at the subwavelength scale.

In the quest for stronger light-matter interactions, it has been shown that light can be confined into even smaller dimensions below 1 nm^3^ when atomic features are present on the nanocavity gap, with a giant enhancement of the EM field in the vicinity of the atomic-scale feature (*15*–*23*). In this context, these nanostructures have been termed as picocavities. The near-field of the LSPs that they support is affected, but not completely determined, by the atomic-scale features present in the nanogap: As previously discussed, the far-field optical response of such cavities depends on the interplay between the light-matter interaction at different length scales ranging from the micrometer to the picometer regime (*20*).

Controllable picocavities are, in general, hard to achieve due to the inherent lack of stability and reproducibility of the atomic-scale features in the nanogap, where very high EM fields emerge. Such control over atomic-scale features is routinely achieved in scanning tunneling microscopy (STM). STM allows for the fabrication, manipulation, and imaging of structures consisting of just a few atoms, where atomic-scale features of metallic surfaces, such as monoatomic step edges, can be studied even at room temperature. Moreover, the gap between the metallic STM tip and the sample surface is known to act as a metallic nanocavity, hosting LSP modes presenting a lateral extension of the order of several nanometers that can be excited by inelastic tunneling electrons. In the presence of atomically sharp structures, such plasmonic cavities acquire the properties of picocavities. Picocavities formed by an atomistic protrusion at the STM tip apex have been proposed to explain the optical response of different systems in many previous works (*24*–*39*). While most of the STM-based picocavities studied so far are produced by attaching an atomic scale object (single atom or single molecule) to the tip apex, a similar behavior has also been found when the atomic-scale feature is attached to the surface (*40*). This last approach has the advantage of a better control on the geometry of the atomistic feature: While the geometry of the tip can hardly be controlled, the geometry of an atomistic feature on the surface can be studied by standard STM imaging. Notice also that, because of the lack of control of the tip geometry, it is difficult to be sure of the presence of atomic-scale features on the tip that would make the gap a picocavity. However, just by repositioning the tip on top of an atomic-scale feature of the substrate surface, we can be confident that the resulting gap is indeed a picocavity.

In this work, we analyze the plasmonic response of a metallic STM picoantenna comprised of a Au tip and a monoatomic height step in a Ag(111) sample. We observe that, upon removal of the electronic factors from the raw optical spectra (*41*), the radiated light into our collection solid angle with the tip placed in close proximity to the step edge can be larger or smaller than that arising from flat terraces, depending on the orientation of the step edge to our light collection angle. This effect can be observed regardless of the tip used to create the picoantenna, independently of its specific symmetry predefining the overall emission direction. The origin of these effects can be traced by comparison with EM calculations. We model the tip as a metallic sphere, and thus, when the tip is placed far away from the step edge, the gap between the tip and the surface can be considered as a nanocavity, since it lacks atomically sharp features. With this model, thus, the angular distribution of the emitted light is isotropic when the tip is far from the step (>3 nm) but it becomes strongly anisotropic when the tip is near the step (±1 nm), forming the picoantenna and leading to larger (lower) intensities being radiated toward the half-plane that contains the upper (lower) terrace. Thus, depending on the orientation of the step edge, the directional nature of the emitted light results in a better/worse alignment to our collection setup (that is fixed), resulting in an increase/decrease of the detected light in the far field, in good correspondence with our experiments. These results demonstrate that a metal STM tip in close proximity to an atomic-scale surface defect can be considered as a realization of a picoantenna, capable of performing its most characteristic function, that is, controlling the directionality of the emitted light. We can envision that this technique could be exploited to tune the direction of light emission of molecules, quantum dots, or other single quantum emitters, opening a window to investigate the optical properties of atom-size objects.

## RESULTS

**Fig. 1.**
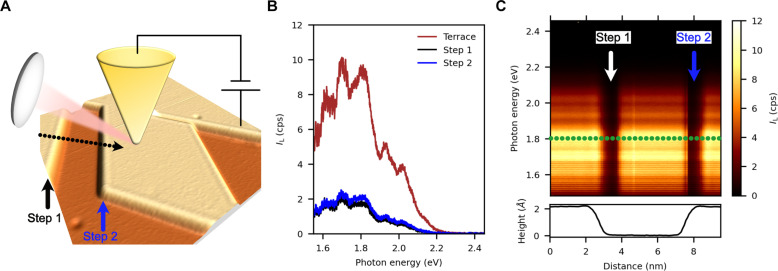
Raw light emission across a monoatomic height step in an STM nanocavity. (**A**) Schematic representation of the experimental setup. A lens near the STM tunnel junction collects the plasmonic luminescence of the tip-sample LSP modes. The black dotted line denotes the trajectory of the tip across two parallel steps (steps 1 and 2, respectively) over the Ag(111) surface. STM image acquisition settings: size = 25 nm × 25 nm, *I*_sp_ = 10 pA, and *V*_sp_ = 1.0 V. (**B**) Raw light emission spectra, *I*_L_, when the STM tip is in close proximity to a monoatomic height step, blue and black curves, step 1 and step 2 in (A), respectively, and over the flat terrace, red curve. Acquisition settings: integration time = 120 s, *I*_sp_ = 350 pA, and *V*_sp_ = 2.6 V. (**C**) Spatially resolved raw light intensity (color-coded) as a function of the photon energy and the tip displacement over the black dotted line in (A). Acquisition settings: integration time/spectrum = 120 s, *I*_sp_ = 350 pA, and *V*_sp_ = 2.6 V; number of spectra = 120. Bottom: height profile corresponding to the trajectory followed by the STM tip in the constant current operation mode demonstrating that the steps are one atom in height.

A schematic representation of the experimental setup is illustrated in Fig. 1A. The light emitted by the radiative relaxation of the LSP modes of the tip-sample cavity excited by inelastically tunneling electrons is collected by a fixed lens. The corresponding STM image shows the surface of a Ag(111) sample with several monoatomic height steps. A typical broadband raw plasmonic spectrum, *I*_L_, of the nanocavity formed by a Au tip and an atomically clean Ag(111) sample is displayed by the red curve in Fig. 1B, where its specific structure (number of subpeaks and intensity ratios) depends on the shape of the STM tip (*42*, *43*). As the black and blue curves in Fig. 1B show, the total emitted light is markedly reduced when the tip is placed near a monoatomic height step edge. Figure 1C displays a series of horizontally stacked spectra traversing two steps across the black dotted line in Fig. 1A, where the color scale represents the raw light intensity. Notice that the scanned line consists of a first step in downward orientation and a second upward, with respect to our collection lens (see Fig. 1A). The color-coded image displays two abrupt light intensity drops that, by comparison with the height profile of the lower panel, can be attributed to the presence of the steps. A comparison of both graphs shows that the measured light intensity reduction is extremely local and occurs over a lateral extension of <1 nm near each step. Notice that the positions where the light intensity features appear with respect to the edges of the steps (see height profile of the bottom inset in Fig. 1C) are independent of the scan direction. Our results are in good agreement with previous reports on the effect of step edges and other atomic-scale defects on STM-induced light emission (STML) spectra (*44*–*47*). Several interpretations have been proposed for such a phenomenon, from variations in the EM coupling between tip and sample at step edges (*45*) to the effect of the modified electronic density of states at the step edges, which would affect the number of available final states for inelastic tunneling (*46*, *47*) and perhaps affect the branching ratio between elastic and inelastic tunneling events (*44*). Because the main observation, i.e., the marked drop of light intensity when the tip is placed near the step edge, could be observed in every step edge regardless of its orientation, none of the previous studies considered the possibility that the light emitted from the picocavity formed when the STM tip is near the monoatomic height step could be anisotropic. Closer inspection of Fig. 1B shows, however, that the intensity drop is not quite the same for step 1 and step 2, a fact that will become relevant later.

As discussed above, raw electroluminescence intensities in STM are determined by not only the optical properties of the nanocavity but also electronic structure factors that consider the relative abundances of empty and occupied states in the tip and sample. Thus, the intensity drop in Fig. 1 might arise from the different electronic structures of flat terraces versus step-edge positions. To unambiguously prove the origin of atomic-like contrast in STML, the electronic structure factor of the measured raw light emission spectra must be properly removed to unveil the pure optical properties of the system, i.e., the radiative component of the photonic density of states (PhDOS). In short, as shown in our previous publication (*41*), to remove the electronic structure factor, one must divide the raw light emission spectra by the rate of inelastic tunneling, *R*_inel_(*ℏ*ω, *V*_b_), which can be experimentally evaluated from the *I*_t_(*V*_b_) curves as *R*_inel_(*ℏ*ω, *V*_b_) ≈ *I*_t_(*V*_b_−*ℏ*ω/*e*), being *I*_t_ the tunneling current, *V*_b_ the bias voltage, *ℏ*ω the photon energy, *e* the electron charge, and *ℏ* the reduced Planck constant. More details about the normalization procedure are included in the Supplementary Materials.

**Fig. 2.**
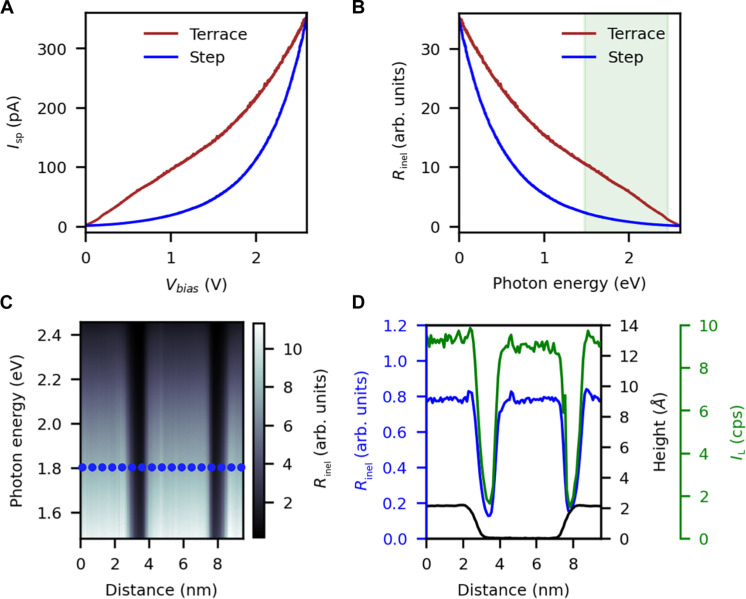
Rate of inelastic tunneling across a monoatomic height step. (**A**) Tunneling current as a function of the bias voltage, *I*_t_(*V*_b_) curve. Red curve: STM tip over the clean terrace. Blue curve: STM tip in close proximity to a monoatomic height step. The tunneling current feedback loop was open during the acquisition. The initial setpoint values determining the tip-sample distance were the same as for the STML curves: *I*_sp_ = 350 pA and V_sp_ = 2.6 V. (**B**) Rate of inelastic tunneling [*R*_inel_(*ℏ*ω, *V*_b_)] obtained from the curves in (A). The green shaded area represents the photon energy window over which the STML data have been acquired. (**C**) Color-coded representation of *R*_inel_(*ℏ*ω, *V*_b_) as a function of the tip displacement over the black dotted line in Fig. 1A. The image corresponds to a vertical stacking of 120 curves. The tunneling current feedback loop was enabled between consecutive curves while the tip moved to maintain a constant current setpoint over the scan line. (**D**) Black curve: height profile of the scanned line in the constant current mode of the STM. Blue curve: *R*_inel_(*ℏ*ω, *V*_b_) across the scanned line at a photon energy of 1.81 eV. Green curve: raw light intensity at a photon energy of 1.81 eV across the scanned line.

Figure 2A shows the measured tunneling current as a function of the bias voltage up to the set point voltage at which the STML spectrum has been recorded when the tip is placed on a flat terrace (red curve) or near a step-edge (blue curve). Because the current used to stabilize the junction is the same in both cases, both curves cross at the set-point voltage. However, the curves are quite different at every other voltage, revealing a substantially different electronic structure. The difference in the electronic structure is probably related with the suppression of the surface state band nearby the step edge (*48*). Once the *I*_t_(*V*_b_) data are converted to *R*_inel_(*ℏ*ω, *V*_b_) (Fig. 2B), it can be observed that the change in curvature implies that the rate of inelastic tunneling is much greater when the tip is over the terrace than the step, due to the lower number of available final states associated with the suppression of the surface state at the step. This result indicates that the STML raw signal near the step will always be smaller than in the terrace just because fewer inelastic events can excite the LSP modes. The evolution of *R*_inel_(*ℏ*ω, *V*_b_) across the two monoatomic height steps over the black dotted line in Fig. 1A is represented in Fig. 2C. As for the raw STML, the color-coded image displays two abrupt drops, which appear at the positions of the steps, with a lateral extension of <1 nm each. Figure 2D shows the comparison of the spatial dependence of *R*_inel_(*ℏ*ω, *V*_b_) (blue curve) together with the raw light intensity at a photon energy of 1.81 eV (green curve) and the height profile of the STM tip (black curve). The topographic features of *R*_inel_(*ℏ*ω, *V*_b_) and the STML raw signal coincide with an accuracy only limited by the STM lateral resolution (~picometer).

**Fig. 3.**
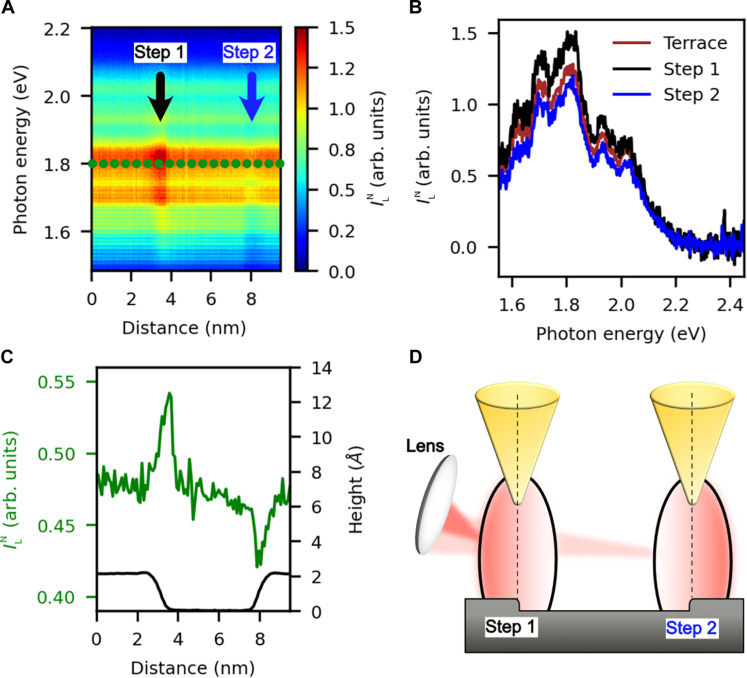
Normalized light intensity across a monoatomic height step. (**A**) Experimentally obtained normalized light intensity, $I_{L}^{N}$ , along the black dotted line in Fig. 1A. After normalization of the raw STML spectra by *R*_inel_, the abrupt changes in light intensity in the proximity of the monoatomic height steps are removed. Still, some small differences remain, originating from local modifications of the true optical properties of the tip-step picocavity. An enhancement/decrease of $I_{L}^{N}$ near the step-edge positions compared to the terrace is observed. (**B**) Spectral distribution of $I_{L}^{N}$ when the STM tip is over the terrace (red curve), near step 1 (black curve), and near step 2 (blue curve). Each curve corresponds to a vertical cut of the color plot in (A). The normalized light intensity of the picocavity formed by the STM tip and the downward step (step 1) is noticeably higher than for the upward step (step 2). (**C**) Green curve: $I_{L}^{N}$ across the green dotted line in (A) at a photon energy of 1.81 eV. Notice that the normalized light locally increases/decreases in the proximity of the two steps referenced to the flat terrace, confined to a lateral extension <1 nm. Black curve: height profile of the scanned line in the constant current mode of the STM. (**D**) Schematic illustration of directional emission for two mirror-symmetric steps. The ellipses represent the distribution of the plasmonic modes of the picocavity formed between the STM tip and a monoatomic height step. The darker red-shaded part of each ellipsis represents a stronger light emission, along the −*x* direction for the left step and +*x* for the right one. Notice that for the left step, the half-space of the collected light contains an upper terrace, whereas for the right step, it contains a lower terrace.

The normalized experimental spectra revealing the pure optical properties of the tip-sample nano(pico) cavity, obtained by dividing the raw light emission by the corresponding rate of inelastic tunneling, are shown in Fig. 3. Figure 3 (A and B) shows that most intensity changes are removed after considering the electronic structure factor. The huge decrease in intensity when the tip is placed near a step edge has disappeared, and all spectra now have similar overall intensities. Thus, the atomically sharp contrast observed in STML of plasmonic systems mainly results from the underlying electronic structure factor, which is inherently convoluted with the raw luminescence. However, a closer inspection of the data shows that small changes in the normalized light intensity, $I_{L}^{N}$ , near the steps remain, which can now be attributed to a purely optical effect. Figure 3A shows a color map of the measured normalized light intensity across the black dotted line in Fig. 1A, traversing the two steps as a function of the photon energy and the tip position. Small changes in intensity are still noticeable: The intensity of $I_{L}^{N}$ of the left step is higher than in the flat terrace, and the right one is lower. When the STM tip is placed more than 1 nm away from the step, the $I_{L}^{N}$ is constant. In the vicinity of the left step, the normalized light intensity increases by 15%, decreasing by 10% when the tip is near the right step compared to the terrace. The observed changes in the normalized light intensity are extremely local, having a lateral extension of <1 nm around each step. To emphasize the locality of the observed changes, Fig. 3C shows the spatial dependence of the measured $I_{L}^{N}$ at a fixed photon energy of 1.81 eV (green dotted cut of Fig. 3A) plotted together with the height profile of the scanned line. It should be noted that the changes in the plasmon intensity can be observed whenever the tip is placed in the proximity of the step edge, even for lateral distances for which vertical repositioning of the tip still does not happen (see fig. S4). Moreover, the effect is independent of the sense in which the STM tip is scanned along the steps, ruling out that the observed effect could be an artifact related to a slow response of our feedback when crossing the step edge.

The fact that the normalized light intensity is appreciably modified by as much as 15% by a step of just one atom height (~2 Å) is a priori unexpected. After ruling out that our observations result from local variations in the electronic density of states by properly removing its contribution from the raw STML spectra, which would modify the number of inelastically tunneling electrons and thus the detected raw light intensity, we can conclude that the effect must be purely optical. Moreover, the intensity differences between both steps must be related to changes in the orientation of the emitted radiation with respect to our collection angle. Notice that, by symmetry, the energy and field intensity of the plasmonic modes of the picocavity formed between our tip and the sample when the tip is near the left step should be the same as when the tip is near the right step. However, the electric field distributions of the plasmonic modes are not necessarily identical, but they should be mirror images of each other through a plane perpendicular to the surface that contains the step edge. The emitted light could thus be different on both sides of such a plane. Because our collection angle is fixed, our experiments give us information about the emitted radiation toward the half-space containing the upper terrace in the left step and toward the half-space containing the lower terrace in the right step, as schematically represented in Fig. 3D. Notice that these observations cannot be ascribed to any asymmetry in the shape of the tip, since the relative orientation of tip and collection angle remains the same for both step edges. Also, it is worth mentioning that the changes in the normalized intensity occur for tip locations for which the vertical position of the tip remains the same, and that they can be observed regardless of the sense in which the scan line is followed, demonstrating that they cannot be attributed to a scanning artifact as in (*49*).

**Fig. 4.**
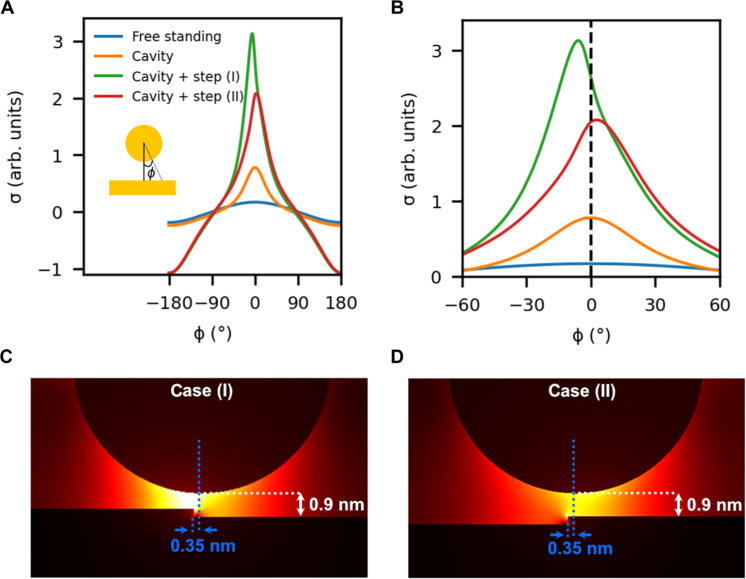
Simulated angular tilt of the induced charge density at the tip and plasmonic field anisotropy due to an atomic height step. (**A**) Calculated surface charge density distribution for different sphere-sample configurations along the spherical perimeter within the plane perpendicular to the step axis, denoted by the angle ϕ. The blue curve, free-standing case, corresponds to an isolated sphere of 5 nm radius as a reference, presenting a cos(ϕ) dependence as expected for a dipolar distribution. The orange curve corresponds to a 5-nm-radius sphere placed at a vertical distance of 0.9 nm from a smooth substrate (in the absence of an atomic step). The green curve corresponds to the surface charge density distribution of the cavity + step system depicted in (C), referred to as case (I). The red curve corresponds to the surface charge density distribution of the cavity + step system depicted in (D), referred to as case (II). Notice that when including the surface, the maximum charge density is larger for a configuration including the step. (**B**) Magnified view of (A) highlighting the shifts experienced by the charge density distributions. The legend is the same as in (A). (**C**) Simulated spatial distribution of the plasmonic field for case (I). (**D**) Simulated spatial distribution of the plasmonic field for case (II).

To gain a deeper understanding of our experimental results, we conducted EM simulations of the STM nanocavity, including the stepped silver surface. We used the same modeling scheme presented in (*41*), based on the finite element Maxwell’s equation solver implemented in Comsol Multiphysics. We modeled the STM tip as a Au sphere of radius 5 nm, placed at vertical distances between 0.5 and 0.9 nm with respect to the relevant terrace and a perfectly vertical step with a height similar to the experimental one. To trace the near-field origin of the experimentally measured anisotropy on the radiation profile, we have analyzed the charge-density distribution on our spherical tip when it is positioned above the step edge (and displaced 0.35 nm away from the origin of the step as in the experiments; see section S2). Gauss law states that the spatial distribution of the induced charge density is given by the discontinuity of the normal component of the electric fields

$$\sigma \propto E_{S^{+}}^{⊥}-E_{S^{-}}^{⊥}$$

where *S*^±^ indicate that the electric field are evaluated outside (+) and inside (−) the metal tip and *E*^⊥^ = **E** · **n**, where **n** is the normal to the tip surface. Figure 4A plots σ evaluated along the perimeter of the 5-nm-radius spherical tip and within the plane perpendicular to the step axis. Four different configurations are considered. The blue line corresponds to the free-standing sphere, and σ presents a cosϕ dependence expected for a purely electric dipole distribution. The orange line shows the charge density in the case of adding a smooth substrate (in the absence of atomic step). Notice that σ(ϕ) is maximum exactly at the tip apex (ϕ = 0). The green and red colored lines, referred to as case (I) and case (II), represent the induced surface charges for two system configurations including a monoatomic step. For case (I), the tip is laterally displaced 0.35 nm from a downward step, while for case (II), the step is in upward orientation. Notably, as shown in Fig. 4B, the maximum charge density is not at the tip apex anymore, but it is displaced a few degrees in the direction toward the upper terrace. This deviation has different sign as the step orientation is opposite. Thus, the simulations show that the presence of the atomistic step gives rise to an angular tilt in the induced charge density at the tip, and therefore in its main multipolar component, the electric dipole one. Moreover, the angular narrowing of σ(ϕ) when the tip is close to the surface (far from the cosϕ dependence of the isolated sphere; see Fig. 4A) indicates that other high-order multipolar contributions are also present. This asymmetric dependence of the charge density is related to the asymmetry of the spatial distribution of the plasmonic modes at 2 eV (Fig. 4, C and D).

**Fig. 5.**
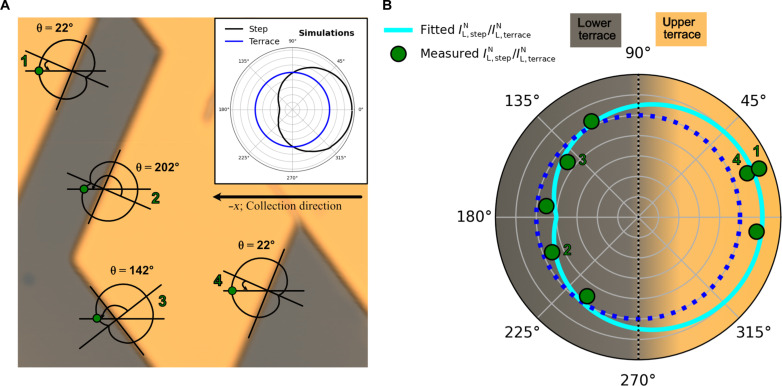
Directionality of plasmonic emission for picoantennas formed by a monoatomic height step. (**A**) STM image of a Ag(111) surface with steps at different orientations. STM image acquisition settings: size = 25 nm × 25 nm, *I*_sp_ = 10 pA, and *V*_sp_ = 1.0 V. The angles formed between the line perpendicular to each step and our collection direction (−*x*) are annotated. Inset: EM simulations of the integrated light emission when the tip (sphere) is placed 0.35 nm away from a monoatomic height step (black curve), and over the flat terrace (blue curve), as a function of the azimuthal angle in polar coordinates. (**B**) Angular distribution of the normalized light (divided by the normalized light at the terrace for comparison with EM simulations). The green dots labeled from 1 to 4 correspond to the integrated normalized light intensity when the STM tip is located near the steps marked in (A), referred to each step’s frame of reference, where the upper terrace is at the right (orange shaded background), and the lower terrace is at the left (gray shaded background). The experimental data as a function of the azimuthal angle defined by the orientations of the steps do not follow a circumference, blue dotted circle representing isotropic emission, implying directional emission. The cyan line is a fitting of our experimental data to a cardioid-like function. The nonlabeled green dots are experimental data at other angles obtained by rotating the sample.

The combination of the angular tilt of the electric dipole induced at the tip and the emergence of stronger multipolar components in its surface charge distribution gives rise to the directional far-field emission. Our EM simulations indicate that when the tip is only 0.35 nm away from the monoatomic step, the radiated power becomes strongly anisotropic, displaying a cardioid shape with a very sharp minimum in the direction perpendicular to the step edge toward the half-space containing the lower terrace (see inset in Fig. 5A). For comparison, and as expected given the symmetry of our model tip, the radiated power is isotropic when the tip is more than 3 nm away from the step edge (see fig. S1 for the criterion for choosing the origin of the step). Depending on the azimuthal angle, the radiated power is higher/lower when the tip is over the terrace/step, yielding a higher/lower intensity along that direction, strongly reminiscent of our experimental results.

Knowing that our collection direction is −*x*, we can calculate the azimuthal angle that corresponds to the emission that we experimentally measure. Figure 5A shows an STM image of a clean Ag(111) surface with steps at different azimuthal angles referenced to the direction along our collection lens. Note that the orientations at which the steps appear are not random and are determined by the high symmetry directions of the crystal. Here, straight step edges were created by gently crashing the STM tip into the substrate with the coarse motor (feedback turned off, step size of 200 nm), after which the surface was reconstructed. We have measured the normalized light intensity across the four steps in Fig. 5A (see fig. S3), displaying three different orientations. Steps 1 and 4 are equivalent, forming an azimuthal angle of 22° referenced to our collection direction. Steps 1 and 2 are mirror symmetric, forming angles of 22° and 202°, respectively. Step 3 forms an angle of 142°. The experimental values of the integrated $I_{L}^{N}$ at 0.35 nm from each step (divided by the normalized light at the terrace) are represented by green-colored dots in Fig. 5B. The remaining not labeled experimental points have been obtained by rotating the sample to access other azimuthal angles (data shown in fig. S4). The gray shaded area represents the lower terrace of a step, and the orange shaded area represents the upper one. Following the theoretically predicted anisotropic shape of the radiated power near the steps, the solid cyan line corresponds to the best possible fit to a cardioid-like function r=r1+(r2−r1)×|sin(π−θ)2| , where *r* and θ are the polar coordinates (radius and azimuthal angle), and *r*_1_ = 0.8 and *r*_2_ = 1.2 are the fitting parameters (radii of the two circumferences forming the cardioid). The blue dotted circumference, representing isotropic emission of a perfectly symmetrical tip, has been included as a guide to the eye to emphasize the directional nature of the emitted light near the steps. Notice the agreement between experiment and theory: When the lower terrace of a step is oriented to our collection (gray shaded area), the datapoints lie inside the blue circumference, whereas when it is the upper terrace (orange shaded area), the datapoints lie outside. This means that the normalized light is higher/lower on the terrace than near the step when the step is in an upward/downward orientation with respect to our collection direction. However, a quantitative comparison between experiment and theory is beyond the scope of our simulations due to the two main simplifications of our model. First, it is assumed that the step is perfectly vertical, not considering the spill out of electrons due to the Smoluchowski effect. Second, we assume a perfectly spherical tip geometry. In addition to the directionality of the emitted light, there is a spectral reshaping of the normalized light emission near the steps due to the enhancement of multipolar components, displaying a sigmoidal-shaped curve with its midpoint at the maximum of the plasmonic emission, in excellent agreement with our simulations (see fig. S2).

## DISCUSSION

The directionality of nanoscale light is a key element for multiple applications relying on quantum light sources. Arrangements of nanostructures can control the directionality of light by far-field interference of different multipolar modes of its constituents when excited by laser irradiation. In nanoantenna-based devices, this can be achieved by engineering the size and shape of the nanoantennas. Alternatively, plasmonic light can be beamed by focusing high-energy electrons (~keV) in cathodoluminescence experiments or low-energy electrons (~eV) in STM on an individual nanostructure, where its geometry and excitation position in the few-nanometer scale also determines the emission direction (*50*, *51*). The mentioned strategies aiming to control the directionality of nanoplasmonic sources rely on imprinting far-field changes of the emitted light by modifying the near field at relatively large scales (few nanometers), compared to the investigations carried out in this work.

Our results demonstrate that monoatomic steps with a height of only 2 Å significantly influence the near-field of plasmonic nanocavities, thereby creating a picoantenna, thus producing nonnegligible effects that can be detected in the far-field. The measured changes in the normalized light of our tip-sample picocavities are restricted to a lateral extension of ~1 nm around each step. Note that the low scanning speed of the STM probe used in the experiments prevents the appearance of *z*-motion artifacts, as may occur in near-field optical microscopies (*49*). The physical origin of the extreme spatial sensitivity found in our experiments is the ultraconfinement of EM fields inside such picoantennas (STM tip-atomic step) and the exponential dependence of the tunneling current on the tip-sample distance, which may be regarded as an extremely local excitation source of LSP modes. EM calculations of the STM nanocavity, including a monoatomic height step and a symmetric tip, show that the experimentally detected changes in the normalized light result from tilting and multipolar reshaping of the tip surface charges near the steps. The tilt in the light emission is such that a 15% increase/reduction in the EM intensity is observed in the far field. Note that real STM tips are not perfectly symmetrical down to the nanometer scale, so that the emission at the terrace is expected not to be perfectly isotropic as in the simulations. However, only the symmetry breaking by the step can cause the far-field directionality observed in our experiments for different steps and different sample orientations. It is important to recall that to access the pure optical properties of the nanocavity, i.e., the radiative PhDOS, one must remove the electronic structure factor from the raw light emission spectra by dividing them by the rate of inelastic tunneling. Otherwise, they are predominantly dominated by the electronic structure factor, hindering the true optical properties of the system.

The outcomes of our investigations suggest that atomic-like defects can play a relevant role in determining the nanometer-averaged response of nanoscale light sources. This may open avenues for designing novel picophotonic devices with patterned structures at the atomic scale. For example, in the light of our work, it becomes interesting the investigation in the directionality of light emission in double or triple step edges, or with a regular array of steps, such as high-miller index crystal surfaces. These systems might show even stronger anisotropic behavior, which could be useful in multiple fields, such as sensing, quantum information, and energy storage, to mention a few.

## MATERIALS AND METHODS

### Experimental setup

The experiments were conducted in a custom-designed low-temperature STM from Omicron operating at liquid helium temperature (4 K) and in ultrahigh vacuum conditions (*P* < 10^−10^ mbar). The STM tips were made of gold. A 0.40-mm-diameter wire was electrochemically etched to sharpen the tips in a 50/50 solution of ethanol and HCl (37% purity). Clean Ag(111) surfaces were prepared by alternating cycles of Ar^+^ sputtering at an energy of 1.5 keV followed by thermal annealing at 500°C. The straight step edges following the high symmetry directions of the substrate were created by gently crashing the STM tip onto the substrate. The electroluminescence signal was first collected by an in situ lens of focal length *f* = 15 mm placed 15 mm away from the tip-sample junction and then guided in free space to an optical spectrometer (Andor Shamrock 500), equipped with a Peltier-cooled charge-coupled device. All the presented spectra are background corrected but are not corrected by the efficiency of the setup.

### Data acquisition

To confront the electroluminescence data with the rate of inelastic tunneling, we recorded STML data and STS curves at each tip position. The feedback loop was kept closed for the STML data to ensure a constant tunneling current during acquisition. For the STS data, the feedback was opened at the same initial setpoint conditions as for STML, and then the bias voltage was swept from the stabilization bias at which the STML data were acquired down to zero. After acquiring an STML spectrum, an STS spectrum at the same position was acquired. To record the next STML spectrum, the feedback was turned on to keep a constant tunneling current while the tip moved. We created a Python script using MATE-for-Dummies (*52*) to govern the routine for data acquisition.

### EM simulations

The numerical calculations were carried out using the frequency-domain finite element solver of Maxwell’s equations implemented in the commercial software Comsol Multiphysics. A full three-dimensional simulation domain was required to accommodate the Au 5-nm-radius nanosphere modeling the STM tip and the Ag flat substrate, decorated by a 50-nm-radius, 0.2-nm-height half disc modeling the atomic-sized step. The height and radius of the total simulation volume was ∼3λ (excluding perfect matching layers). A conformal mesh distribution accounted for the EM field propagation from the picometric antenna gap, driven by a point-like dipole at its center to the circular-shaped area where the time-averaged Poynting vector was integrated. This was located ∼1λ away from the dipolar source, and its dimensions were set to mimic the solid angle covered by the collection lens in the experiments. The convergence of numerical results against the mesh size and distribution was checked. All permittivities were taken from (*53*).
