# Supplementary material for: Directional picoantenna behavior of tunnel junctions formed by an atomic-scale surface defect
Source: Sci Adv. 2024 Sep 25;10(39):eadn2295. doi: 10.1126/sciadv.adn2295 (PMC11423879; doi:10.1126/sciadv.adn2295)
Supplement: Supplementary file 1 — Supplementary Text Figs. S1 to S5 [file sciadv.adn2295_sm.pdf]

Science Advances
AAAS

# Supplementary Materials for

## Directional picoantenna behavior of tunnel junctions formed by an atomic-scale surface defect

David Mateos *et al.*

Corresponding author: Alberto Martin-Jimenez, alberto.martinj@imdea.org

*Sci. Adv.* **10**, eadn2295 (2024)
DOI: 10.1126/sciadv.adn2295

**This PDF file includes:**

Supplementary Text
Figs. S1 to S5

1. Normalization procedure

As described in our earlier publications (*25*, *41*), for a nano(pico)cavity supporting a localized plasmonic mode of energy $h\nu$, the excitation efficiency by inelastic tunneling electrons can be expressed as

$$(1)\ R_{inel}(\hbar\omega, V_B) \propto \int \rho_T(E + \hbar\omega - eV_B) f(E + \hbar\omega - eV_B)\rho_S(E)[1 - f(E)]T_{\mathrm{inel}}(E, h\nu, eV_{\mathrm{B}})dE,$$

where, $\rho_T$ and $\rho_S$ are the densities of electronic states of tip and sample respectively, $f$ is the Fermi-Dirac distribution and $T$ is the transmission factor. As long as the energies involved are more than a few times larger than the thermal energy $K_B T$, the previous expression can be simplified by

$$(2)\ R_{inel}(\hbar\omega, V_B) \propto \int\ \rho_T(E + \hbar\omega - eV_B)\rho_S(E)T(E, V_B, \hbar\omega)dE$$

Following the traditional Bardeen approach, elastic tunneling currents between two metallic electrodes can be calculated as:

$$I_t(V_B) \propto \int^{eV_B} \rho_T(E - eV_B)\rho_S(E)T(E, V_B)dE$$

By noticing the similarities between expressions (1) and (2), we can conclude that

$$R_{inel}(\hbar\omega, V_B) \propto I_t(V_B - \hbar\omega/e)$$

For photon energies below $eV_B$. $R_{inel}$ is the electronic structure factor used to normalize the raw STML data for obtaining the pure optical properties, $I_L^N(\hbar\omega, \mathrm{V_B})$.

2. Criterion used to define the tip lateral position for comparing the normalized light near the steps at different azimuthal angles

When the STM tip traverses a monoatomic height step in the constant current operation mode the tip-sample distance may vary due to additional lateral contributions to the tunneling current (used as the feedback mechanism) from both sides of the step. Therefore, to exclude any possible scanning artifact due to variations in the gap size when the tip is traversing the step, we place the STM tip 0.35 nm away from the center of the step, indicated by the black dashed line in Fig. S1, where the near field is still influenced by the atomic step, but now the tip-sample distance is the same as for the terrace when the tip is away > 3 nm. This is the criterion used to compare the experimental data of the polar plot of Fig. 5B for steps at different angles.

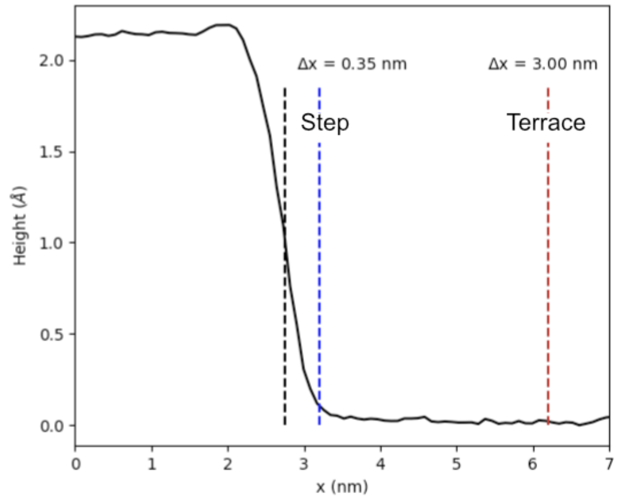


**Fig. S1. Definition of lateral distance to the step for comparing the normalized light near the step and terrace**. Height profile over a monoatomic height step acquired in the constant current mode of the STM. The dashed black vertical line at the center of the slope represents the origin chosen for referencing the lateral distance between the STM tip and the step. When Δx is beyond 3 nm (dashed red vertical line), the effect of the step is negligible, and the tip-sample cavity can be considered a nanocavity. For a distance of Δx=0.35 nm (dashed blue vertical line), the monoatomic step still influences the electromagnetic response of the picocavity formed by the STM tip and the step, but now the tip-sample distance is the same as for the lower terrace. By locating the STM tip at these two relative positions to the center of a step we can safely compare the normalized light intensity near the step and terrace, minimizing possible artefacts that may be present due to differences in the tip-sample height at shorter lateral distances to the step.

## 3. Spectral reshaping of plasmonic emission near a monoatomic height step

To discriminate if the modifications of the normalized light near the step only change the global intensity due to a directional picoantenna behavior or whether the spectral distribution has also changed, we define a spectrally-resolved contrast factor as $\Delta I_L^N = \left(I_{L,step}^N - I_{L,terrace}^N\right)/I_{L,terrace}^N$. Since the modelling parameters of the sphere-surface nanocavity (5 nm radius sphere and 0.9 nm tip-sample distance) have not been optimized to yield a plasmonic resonance in the same energy window as the experimental data, we define a relative photon energy axis, $\Delta E_{phot}$, to quantitatively compare theory and experiments. This energy scale, $\Delta E_{phot} = E_{phot} - E_{cent}$, references the photon energy to the centroid of the spectrum , $E_{cent}$, that will be different four our experiments and simulations.

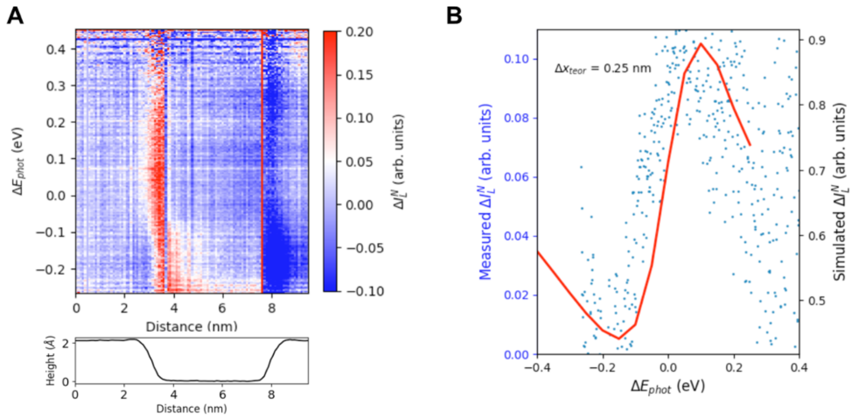


**Fig. S2. Spectral reshaping of the normalized light intensity near a monoatomic height step. (A)** Spectrally-resolved contrast factor of the normalized light intensity, $\Delta I_L^N$, as a function of the relative photon energy, $\Delta E_{phot}$ along the black dotted line in Fig. 1A. The contrast plot highlights the spectral reshaping of the normalized light when the tip is in close proximity to each step. The higher/lower normalized light near the two steps is now visualized as a positive/negative quantity in the contrast map. **(B)** Spectral distribution of $\Delta I_L^N$ when the STM tip is 0.35 nm from the left step. Blue dots: experimental data. Red curve: EM simulations. Not all wavelength components are enhanced in the same way. The contrast plot has a sigmoidal shape with its center at the photon energy of the maximum of the plasmonic emission, $\Delta E_{phot} = 0$.

4. Normalized light along other steps

Normalized light emission along the four steps of Fig. 5A as a function of the STM tip position and photon energy. The right-hand side panels are the corresponding contrast factors, $\Delta I_L^N$, to highlight the spectral reshaping near each step.

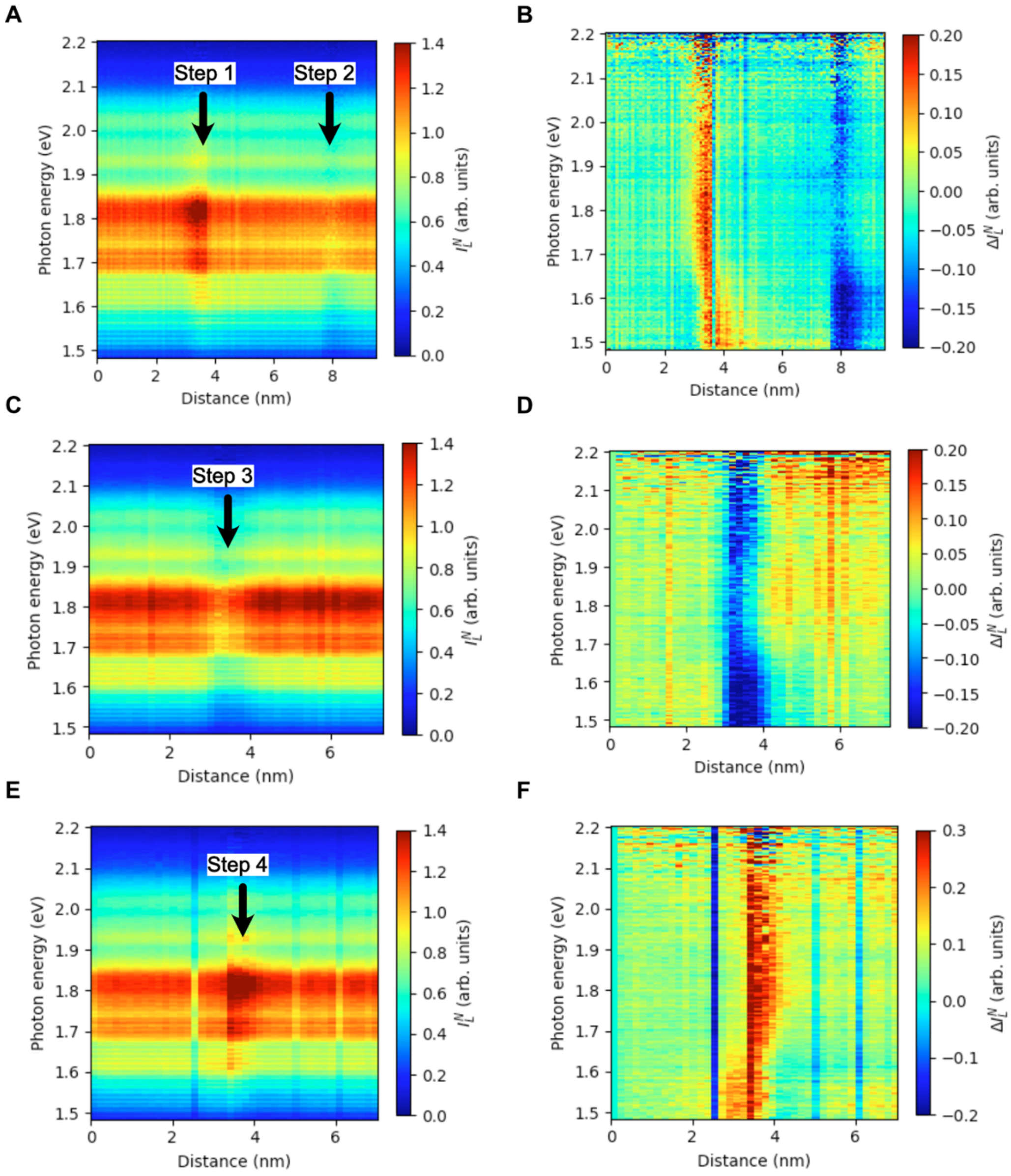


**Fig. S3. Normalized light intensity for different orientation steps. (A)** $I_L^N$ as a function of the photon energy and tip position along steps 1 and 2 in Fig. 5A. **(B)** Corresponding contrast factor, $\Delta I_L^N$, of (A). **(C)** $I_L^N$ as a function of the photon energy and tip position along step 3 in Fig. 5A. **(D)** Corresponding contrast factor, $\Delta I_L^N$, of (C). **(E)** $I_L^N$ as a function of the photon energy and tip position along step 4 in Fig. 5A. **(F)** Corresponding contrast factor, $\Delta I_L^N$, of (E).

## 5. Experiments with different STM tips

Here we present additional experiments with different STM tips confirming the robustness of the picoantenna behavior near the monoatomic height steps. Tips 1 and 3 were made from a 0.40 mm diameter gold wire, and Tip 2 from a 0.25 mm gold wire. The morphology of tip 3 was changes by crashing the STM tip with the piezo motor. All tips display different plasmonic modes as evidenced by their spectra.

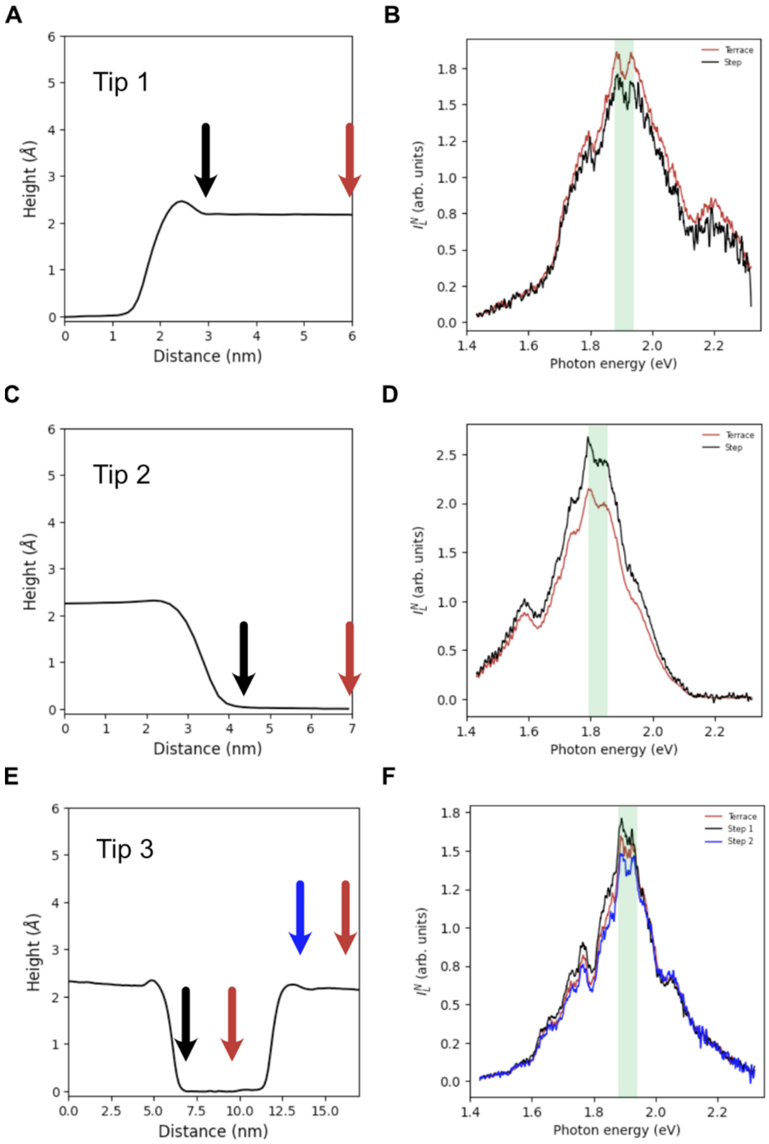


**Fig. S4. Experiments with different tips**. **(A)** Height profile of an ascending monoatomic step acquired in the constant current operation mode of the STM with tip 1. **(B)** Normalized light intensity at the positions marked by the vertical arrows in (A) with tip 1. Tunneling parameters: Isp =350 pA, Vsp = 2.6 V. Acquisition time/spectrum = 30 s. **(C)** Height profile of a descending monoatomic step with tip 2. **(D)** Normalized light intensity at the positions marked by the vertical arrows in (C) with tip 2. Tunneling parameters: Isp =350 pA, Vsp = 2.6 V. Acquisition time/spectrum = 30 s. **(E)** Height profile of two steps acquired in the constant current operation mode of the STM with tip 3. **(F)** Normalized light intensity at the positions marked by the vertical arrows in (E) with tip 3. Tunneling parameters: Isp =350 pA, Vsp = 2.6 V. Acquisition time/spectrum = 30 s.

6. Simulations with spheres of different radii

Simulations of the plasmonic response for tip-step picocavities formed by spheres of different radii. We have used 5 nm and 10 nm spheres, red and black curves, respectively. The gap was 0.9 nm in both cases. As expected for a larger radius sphere, the plasmonic spectrum red-shifts, which is seen as a shift of the spectral reshaping factor in Fig. S5 A. Fig. S5 B shows the spectral reshaping factor referred to the maximum of each plasmonic

resonance, where it can be observed that the tip radius does not affect the sigmoidal-like behavior of the contrast factor curve.

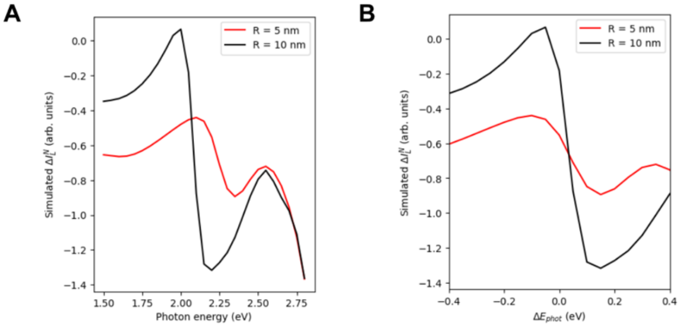


**Fig. S5. Simulations with spheres of different radii**. **(A)** Simulated spectrally resolved contrast factors, $\Delta I_L^N$, for a sphere of 5 nm radius (red curve) and a sphere of 10 nm (black curve) placed at a vertical distance of 0.9 nm from a perfectly vertical step of 0.2 nm height and a lateral distance of 0.35 nm as a function of the photon energy. The red-shift of the black curve is due to the larger sphere radius. **(B)** Simulated spectrally resolved contrast factors referenced to the maximum of each plasmonic emission, that is 2.20 eV for the 5 nm sphere, and 2.05 eV for the 10 nm sphere. The sigmoidal-like behavior observed in our experiments (Fig. S2) is maintained, not depending on the specific tip radius used in the simulations.

## 7. Influence of scanning direction

The scanning speeds when traversing a step are very low because each experiment records the plasmonic emission and inelastic tunneling rate at each lateral position of the tip. The scanned lines consist of the order of 100 points each, resulting in acquisition times of tens of minutes per experiment. Therefore, the feedback response is accurate enough so that there is no noticeable difference between forward and backward scan. Fig. S3 displays two identical downward steps, steps 1 and 4, respectively, that have been acquired by scanning the STM tip in opposite sense (step 1 in forward direction and step 4 in backward). Notice that both experiments display the same behavior, i.e., the normalized light intensity is larger when the tip is near the step due to its orientation with respect to our collection lens (the upper terrace lies at the left-hand side), ruling out that the directionality behavior is due to or influenced by the scanning direction.

## 8. Influence of tip-height variation at the step

When the tip is right on top of the step, its vertical distance changes while scanning, which might affect the optical signal. A change in the tip's vertical positioning might lead to variations in the LSP modes which are not just due to the presence of the step. However, as shown in Fig. S4, the effect of the step-edge on the collected light intensity can be found for separations from the step-edge for which the tip is still not changing its vertical position respect to the flat terrace.
